# AdCOFE: Advanced Contextual Feature Extraction in conversations for emotion classification

**DOI:** 10.7717/peerj-cs.786

**Published:** 2021-12-09

**Authors:** Vaibhav Bhat, Anita Yadav, Sonal Yadav, Dhivya Chandrasekaran, Vijay Mago

**Affiliations:** Department of Computer Science, Lakehead University, Thunderbay, Ontario, Canada

**Keywords:** Emotion recognition, Chatbots, Dyadic conversation

## Abstract

Emotion recognition in conversations is an important step in various virtual chatbots which require opinion-based feedback, like in social media threads, online support, and many more applications. Current emotion recognition in conversations models face issues like: (a) loss of contextual information in between two dialogues of a conversation, (b) failure to give appropriate importance to significant tokens in each utterance, (c) inability to pass on the emotional information from previous utterances. The proposed model of Advanced Contextual Feature Extraction (AdCOFE) addresses these issues by performing unique feature extraction using knowledge graphs, sentiment lexicons and phrases of natural language at all levels (word and position embedding) of the utterances. Experiments on emotion recognition in conversations datasets show that AdCOFE is beneficial in capturing emotions in conversations.

## Introduction

There have been significant advancements in conversational AI research, where emotion recognition in conversations has been acknowledged as one of the crucial areas of research. The task of identifying or predicting the emotion of each dialogue in any conversation is called emotion recognition in conversations (ERC) (Hazarika et al., 2021). This identification or prediction of emotions in conversation is an essential step in any conversation understanding applications. ERC has proven to be indisputably important in real-time dialogue systems like emotion-aware chat agents (Li et al., 2020), visual question answering (Tapaswi et al., 2016), and many other applications where the potential of ERC is immense.

Various virtual digital agents and chatbots are available on different websites which are used by a large number of online users. These chatbots rely mainly on the ability to generate responses depending upon the users’ emotions. The bots need to detect these emotions and sentiments in order to provide emotionally coherent and empathetic responses by avoiding any inappropriate response (Miner et al., 2016). In conversational agents, models are trained to understand human emotions in a conversational context (Zhong, Wang & Miao, 2020). Emotion detection has also been providing effective results in the area of opinion mining over a chat history, social media threads, and debates.

There is a wide range of other applications which can utilize ERC. For instance, while providing customer service on a social media platform like Twitter (https://twitter.com/), ERC models can provide quick responses to the issues raised through numerous tweets (Chatterjee et al., 2019). Models can prioritize the tweets according to the emotion in the content and respond to the messages with emotion class of angry or upset providing them maximum user satisfaction (Chatterjee et al., 2019). Additionally, ERC can help applications to prevent sending or posting of messages by tagging them as bullying or threatening, thus warning online users before sending such messages.

The different ERC models proposed in the existing research provide the sentiment classification of the utterances in a dyadic conversation (a conversation of two participants). These models have not captured some of the key parameters which need to be attended to in order to recognize emotion in conversations. Some models do not perform ideal feature extraction, or some models lack the minute attention which must be given to significant tokens in an utterance. In AdCOFE, we intend to address such issues by extracting advanced contextual features in the feature extraction layer prior to passing them through the main classification model. The key methodological highlights of our research work are: (a) Utilizing knowledge graphs for an advanced contextual understanding of the sentences, (b) employment of sentiment lexicons for adding context-based emotional features to the sentences, and (c) use of a simple and elegant pretrained model which is efficiently fine-tuned for the purpose of ERC. With these advances, the results achieved are better as compared to the state-of-the-art models and are comparable to the latest models on the Interactive Emotional Dyadic Motion Capture (IEMOCAP) dataset.

The remainder of the paper is organized as follows: The related Work section provides insights of previous research work and the progress in ERC; the Methodology section describes the dataset used, discusses our proposed approach, algorithm and provides details on experimental setup; the Results and Discussion section reports the results and provides a comparative analysis with both the baseline as well as the latest ERC models; finally, we conclude the paper signifying the distinctive approach implemented and also mention the future work to further enhance the classification model.

## Related Work

Emotion recognition in conversation has gained immense popularity in a very small span of time due to its widespread applications. As described in the introduction section, ERC mainly consists of context representation, capturing the impact of the contextual information from previous utterances, and extracting emotional features to perform classification. To achieve this contextual modeling in either textual or multimodal settings different deep learning-based algorithms are being used. Poria et al. (2017) used RNNs for multimodal emotion recognition which propagated contextual and sequential information to the utterances. Majumder et al. (2018) enhanced these deep learning models further by incorporating party and global states in the recurrent models for modeling emotional dynamics. The party states are used to keep track of each speaker while the global state keeps account of the context. Ghosal et al. (2020) utilized the speaker-level context encoding like Majumder et al. (2018), using a two-step graph convolution process. Along with speaker context, sequential context was extracted to overcome the issue of context propagation in the DialogueRNN model (Majumder et al., 2018). However, RNNs fail to take into consideration the dependencies between two utterances in a conversation causing loss of long-range contextual information in a dialogue.

Jiao et al. (2019) proposed a hierarchical Gated Recurrent Unit (GRU) framework with self-attention and feature fusion (HiGRU-sf) model to capture long-range contextual information among words and utterances for ERC. Further, Jiao, Lyu & King (2019) proposed an Attention Gated Hierarchical Memory Network (AGHMN) where the Hierarchical Memory Network (HMN) is responsible for enhancing features at utterance level and memory bank that can be used for contextual information extraction. The two-level GRU layer of HMN is responsible for modeling the word sequence of each utterance called the utterance reader, and the other layer adopts a BiGRU structure to capture historical utterances. In addition to this HMN network, an Attention GRU (AGRU) is added which promotes comprehensive context and retains positional and ordering information of the utterance. Li et al. (2020) created a generalized neural tensor block (GNTB) followed by two emotion feature extractor (EFE) channel classifiers to perform context compositionality and sentiment classification respectively. Similar to bidirectional long short term memory (BiLSTM), two emotional recurrent units (ERU) are utilized for forward and backward passing of the input utterances after which the outputs from these forward and backward ERUs are concatenated for sentiment classification or regression (Li et al., 2020).

Additionally, there have been further efforts in models to give variable importance to each word in an utterance and include information from speaker context into the model. Ragheb et al. (2019) suggested an attention-based modeling process to address the problem of equal weight to each token in a sentence. Ragheb et al. (2019) utilized a three-layered BiLSTM model, which was trained using Average Stochastic Gradient Descent (ASGD), and a self-attention layer to extract features enriched with contextual information. Lately, Xing, Mai & Hu (2020) proposed an Adapted Dynamic Memory Network (A-DMN) which models self and inter-speaker influences individually which was not incorporated effectively in previous models. A-DMN consists of a global RNN to capture the inter-speaker influence of the utterances in a dialogue along with individual RNNs for each speaker to capture their self-influence. These captured contexts are used to update the memory with multiple passes to create a refined representation to capture the emotion. Overall, due to the sequential nature of the data, the use of recurrent neural networks is in abundance. Moreover, it is pivotal that the speaker context, sequential context, and the emotions of the previous utterances are passed throughout the model.

Recently, there has been development in the concept of utilizing graphs to provide solutions for recognizing emotions. Xu et al. (2020) proposed EmoGraph, which utilizes graph networks to identify the dependencies among various emotions. The co-occurrence statistics in between different emotion classes are used to create a graphical neural network, taking each emotion class as a node. This helps the network in extracting features from the neighbours of each emotion node. Ishiwatari et al. (2020) highlighted that the different graph-based neural networks miss out on the sequential information of the conversation. To overcome this issue, a relational position encoding is proposed in the Relational Graph Attention Network (RGAT) which would reflect the speaker dependency and sequential information by applying positional encoding in the relational graph structure.

Another approach to capture emotions in conversation is by transfer learning model which utilizes a pre-trained hierarchical generative dialogue model, trained on multi-turn conversations (Hazarika et al., 2018). The knowledge acquired from dialogue generators is transferred to a classification target model. The classification model uses the learned context and parameters from the pre-trained model output and projects it to the label-space (Hazarika et al., 2018) which helps it to identify the emotion for the given sentence. Furthermore, Zhong, Wang & Miao (2020) used a Knowledge-Enriched Transformer for transfer learning using pre-trained weights. In the suggested model, all the tokens in the given utterance of the conversation are converted into a specific vector called Valence Arousal Dominance (VAD) (Mohammad, 2018). The conceptual knowledge is characterized using Dynamic Context-Aware Affective Graph Attention and the contextual knowledge is obtained using a Hierarchical self-attention. Moreover, a context-aware concept-enriched (Bahdanau, Cho & Bengio, 2014) response representation for a given conversation is learned. Different pre-trained models and transformers have been implemented in the field of ERC to increase the computational speed as well as getting better results. Also, the usage of attention-based models in ERC is evident.

On examining the above mentioned models we identify the following research gaps: (a) loss of contextual information in between two dialogues of a conversation, (b) failure to give appropriate importance to significant tokens in each utterance, and (c) inability to pass on the emotional information from previous utterances. We attempt to address these shortcomings by the model proposed in this article.

## Methodology

### Datasets

The IEMOCAP dataset collected at the SAIL lab at the University of Southern California is designed to capture expressive human communication and synthesize different aspects of human behaviour (Busso et al., 2008). The dataset outlines a dyadic conversation among 10 different speakers. The dataset is an acted, multimodal database that contains 12 h of audio-visual data including speech, motion capture of the face, and text transcriptions. The recorded sessions containing the dialogues were manually segmented based on the dialogues turn level and multi-sentence utterances were split as single turns. The emotion labels assigned to the *corpus* were done using different annotation schemes which captured the emotional aspect of the dialogues. The most popular assessment schemes used were: discrete category-based annotations (*i.e*., labels such as happiness, anger, and sadness), and continuous attribute-based annotations (*i.e*., activation, valence, and dominance). Six human evaluators were asked to assess the emotional categories for the *corpus*. The interactions recorded were to be as natural as possible, hence the evaluators came across more than the normal set of emotions which led to ambiguous results. As a final trade-off, the utterances were categorized into the following emotions: happiness, sadness, neutral, anger, excitement and frustration. Another inappropriate approach which undermines the intensity level of the emotions demonstrated by the utterances would be to use attributes like valence, activation (or arousal), and dominance. Hence, in order to provide insights about both how people displayed emotions and how they can be automatically recognized we need both types of emotional descriptions. This *corpus* is widely used in understanding expressive human communications and contributes to a better emotion recognition system.

In this article, we use the preprocessed IEMOCAP dataset provided by Ghosal et al. (2020). Initially, the sentences are given as a list containing all the sentences along with information on the length of each conversation. There are a total of 96 conversations in the training set and 31 conversations in the testing set containing 4,699 sentences and 1,624 sentences respectively. All of the conversations are dyadic conversations. Each utterance varies in length. Similarly, each conversation varies in length as well. The labels are numbered from 1–6 for the six possible emotion outputs of the IEMOCAP dataset. Each sentence has a specific label assigned to it according to the emotion it depicts. Table 1 shows the distribution of each label in the dataset.

**Table 1:** Distribution of labels in the IEMOCAP dataset.

Emotion	Count
Happy	376
Sad	764
Neutral	1,080
Angry	749
Excited	520
Frustrated	1,210

In addition, a multimodal multi-party dataset for emotion recognition in conversation (MELD) dataset (Poria et al., 2018) is also used. The MELD dataset is an extension of the EmotionLines dataset consisting of the dialogues from the television show Friends. The dataset contains seven possible labels for each utterance: anger, disgust, fear, joy, neutral, sadness, and surprise.

For our purpose, we use the dyadic version of the MELD dataset which is an enhanced version of MELD specifically for textual conversations which are dyadic in nature. The dataset was developed in 2019 for the specific purpose of having dyadic conversations only. In this dataset, the training set has 12,840 sentences while the test set has 3,401 sentences. The labels are initially in the string format which are then replaced with numbers ranging from 0 to 6 corresponding to the seven labels previously mentioned. Table 2 shows the distribution of each label in the MELD dataset.

**Table 2:** Distribution of labels in the MELD dataset.

Emotion	Count
Anger	1,500
Disgust	364
Fear	338
Joy	2,312
Neutral	5,960
Sadness	876
Surprise	1,490

### Problem statement

Let there be *n* utterances in a conversation (*C*) represented as *u*_*n*_. Let the two speakers in the dyadic conversation be denoted by ${p_1}$ and ${p_2}$. Let there be a mapping *S* between the speaker and utterance denoted by $S:{p_i} \to {u_j}$ where *i* ∈ {1,2} and *j* ∈ [1,*n*] indicating which speaker uttered the given sentence in the conversation. We have a set of emotions *E*, which has values for six emotions (Happy, Sad, Neutral, Angry, Excited and Frustrated) ranging from 0–5. Our task is to identify the emotion of each mapping *S* in the conversation and this can be extended as a variable that varies over a range.

### Data preprocessing

We use the IEMOCAP and MELD dataset for obtaining results from our proposed algorithm. The labels for each sentence are either of the following: Happy, Sad, Neutral, Angry, Excited, and Frustrated for IEMOCAP (Busso et al., 2008) and Anger, Disgust, Fear, Joy, Neutral, Sadness and Surprises for MELD. For preparing the data to pass through the respective layers of the model, two different processes of data preprocessing methods are used. At first, the data is preprocessed using the NLTK library to remove stop words and punctuation and then tokenized to extract contextual information using ConceptNet Web API (Speer, Chin & Havasi, 2016) and Python GET requests. This preprocessed data is further passed through the sentencepiece tokenizer and BERT (Devlin et al., 2019) preprocessor to obtain a fine-tuned data for the *A Lite BERT* (ALBERT) (Lan et al., 2019) pretrained model for classification.

### Proposed method

Given transcripts of long-range recorded dyadic conversations, the ERC system aims to identify the emotions in each utterance and label them into appropriate emotion categories. The major task here is to capture long-range context from conversations and passing the emotions from one utterance to another. As the *corpus* consists of multiple words with different meanings based on the context, the task of the ERC system is to identify the emotion based on their position and importance in an utterance.

Keeping these shortcomings in mind, we propose AdCOFE (Implementation available at https://github.com/VBhat97/AdCOFE) which has the following characteristics:

• Context-dependent feature extraction with importance to word and position embedding in an utterance.

• Contextual enrichment to existing sentences by using knowledge graphs and emotion lexicons.

• Incorporation of pretrained transformer models for final emotion classification.

**Figure 1:**
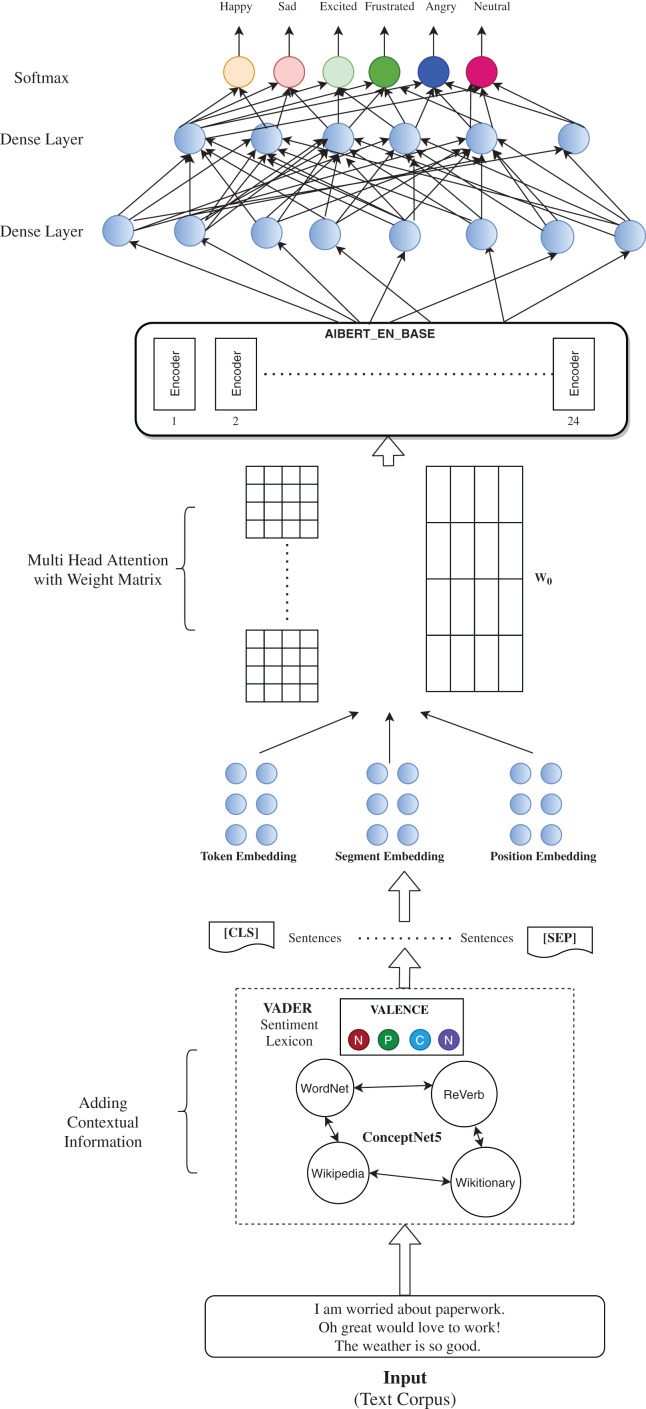
Proposed model architecture.

The architecture in Fig. 1. Summarizes the flow of our proposed method. The IEMOCAP *corpus* consists of long recorded dyadic conversations which serve as the input to our model. The initial step of AdCOFE is to enhance feature extraction for better classification. This is done by using basic preprocessing steps. In the next steps for contextual enrichment, we make use of ConceptNet API and VADER emotion lexicons which analyze the context and add more contextual information to the existing *corpus*. These enriched sentences are then tokenized into various embeddings with special attention to their positions in the utterance. The enhanced context enriched text *corpus* is then trained using the ALBERT model which then outputs a 1 × 786 dimensional matrix which is passed to the Fully Connected Dense Layers. At the end, we have a *softmax* layer which classifies the sentence according to one of the six emotion labels.

#### Initial feature extraction

The text *corpus* involves a set of dyadic conversations which involves two participants. Each participant has a set of utterances that are associated with a particular emotion. The initial step involves data preprocessing in order to achieve the sentences in the required format whereon we can analyze them. After this, the main focus is on context extraction which our model AdCOFE handles by passing the utterances from a single speaker in a batch of sentences. This step ensures that the emotion is carried throughout the model while keeping the length of conversations constant. This helps in the easy classification of emotions even in long-range conversations.

**Figure 2:**
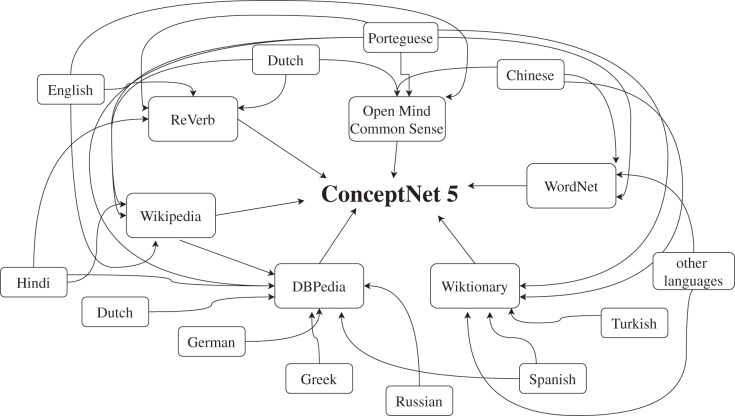
Example of the ConceptNet graph.

**Figure 3:**
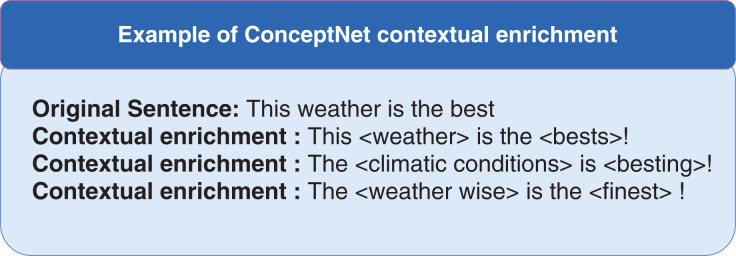
Example of ConceptNet contextual enrichment.

The next steps deal with context enrichment to already existing text *corpus*. We employ a ConceptNet model proposed by Speer, Chin & Havasi (2016) (example shown in Fig. 2.) which is a knowledge graph that connects words and phrases of natural language (terms), weighted edges (assertions). ConceptNet’s knowledge graph is compiled from various well-known sources and consists of 21 million edges, over 8 million nodes, 1,500,000 nodes for the English language itself, and 83 languages with at least 10,000 nodes. Each word in the vocabulary is represented as a node and the edges depict how trivial or non-trivial the context between the two words is. It uses a closed class of selected relations such as IsA, Used For, and Capable Of for representing the edges. (Speer, Chin & Havasi, 2016) For our model we use ConceptNet 5.7 to extract connected words to the given words in the utterances. ConceptNet Web API (http://api.conceptnet.io/) is used for getting the required contextual information through GET requests. After arranging the sentences as mentioned in the previous step, each sentence is preprocessed using the ConceptNet preprocessing techniques des. The tokens are then passed to the ConceptNet Web API to retrieve the top related words as a response to the request based on the ConceptNet knowledge graph. These tokens are then replaced in the sentences and added to the training data. An example is shown in Fig. 3. In this example the original sentence is “This weather is the best!” We first remove the stopwords from the sentence which is a part of the preprocessing step. The tokens <weather> and <best> remain and these tokens are passed to the ConceptNet API. The top three responses from the ConceptNet API for the word <weather> are <weathers>, <climate conditions> and <weather wise> and for <best> are <bests>, <besting> and <finest>. Hence, these words are replaced in place of the original tokens and added to the training dataset.

The training data then contains both the original sentence as well as the contextually enriched sentences from ConceptNet. This enhances the contextual information in the training data by adding similar sentences. Thus, contextual features are obtained based on the knowledge graph of ConceptNet (ConceptNet API : http://api.conceptnet.io/).

In the next step VADER (Wilson & Hernández-Hall, 2014), a simple rule-based model for sentiment analysis is used. The sentiment lexicon uses a combination of rules to generate lexical features which closely display the sentiment intensity of a particular sentence. VADER (Wilson & Hernández-Hall, 2014) follows a four-step methodology for sentiment analysis. In the first step, it constructs and validates a valence-aware sentiment lexicon by including sentiments from various word-banks, adding certain features to it, and then rating these features from a scale of “Extremely Negative” to “Extremely positive” with allowance for “Neutral”. In the next step, it identifies generalizable heuristics humans use to assess sentiment intensity in the text. This included analysis of 800 tweets (400 positives and 400 negatives) with Pattern (a web mining model) as well as through human intervention. The next step controls experiments to evaluate the impact of grammatical and syntactical heuristics followed by ground truth in multiple domain contexts. The features obtained are rated on four scales from Negative, Positive, Neutral to Compound. These lists of features are then used to determine the sentiment of the utterance based on a threshold score and are added with the context enriched sentences obtained in the previous step. The combination of contextual information obtained as a result of the ConceptNet API and the sentiment lexicon of VADER is then passed on to the ALBERT classification model.

#### Emotion classification model

For Emotion Classification, ALBERT is used for self-supervised learning of language representations. ALBERT enhances the AdCOFE model by identifying bidirectional context, emotions of the sentences where the same words are used in different contexts, and acknowledging the importance of certain words in the sentence. For example, for a given sentence from the dataset: “Eww! It’s the Mattress King” the model is able to identify the emotion of “It’s” as disgusting based on the word “Eww!” and its positioning. Results of the ALBERT model are better as compared to the original BERT model (Devlin et al., 2019) in ERC tasks. This is due to the appropriate architectural choices taken in the ALBERT model which helps to overcome the issues faced by the traditional ERC models. These specifically include:

• **Factorized embedding parameterization:** The Word Piece embedding used in BERT (Devlin et al., 2019), XLNet (Yang et al., 2019), and RoBERTa (Liu et al., 2019) have a size that is tied with the size of the hidden layer. This is proved to be suboptimal from both modeling and practical perspective (Lan et al., 2019). From the modeling point of view, the untying of the size of WordPiece embeddings and hidden layer embeddings makes efficient usage of total parameters, as in ERC these context-dependent representations have great importance. From a practical point of view, due to the large vocabulary of the sentences, the factorization of embedding parameters gives us an added advantage on the execution time as compared to BERT. Moreover, it also keeps in check the attention given to different words of a sentence which is pivotal as well.

• **Cross-layer parameter sharing:** ALBERT uses a cross-layer parameter sharing strategy which helps on stabilizing the network parameters (Lan et al., 2019). In terms of ERC, this helps in finding the best options for tuning the hyperparameters much quicker as compared to BERT. Although it does not affect the hyperparameters such as number of epochs and learning rate, this model architectural choice in ALBERT helps to reach them efficiently.

• **Inter-sentence coherence loss:** ALBERT follows sentence-order prediction (SOP) loss which does not take into consideration topic prediction but is heavily based on inter-sentence coherence (Lan et al., 2019). This helps to improve the performance of multi-sentence encoding tasks. In terms of ERC, it becomes extremely important to take into account the coherence between the sentences as they are part of the same conversation. The three back-to-back utterances of a specific speaker are obviously connected and important for the speaker to be reasonable, thus taking into account coherence between the utterances plays an important role. As a result, the combination of taking context from the words in an efficient way as well as the coherence in between the sentences together helps to select ALBERT over other BERT models.

The proposed model, according to Algorithm 1 uses a pre-trained ALBERT_EN_BASE model which serves the purpose of overcoming the shortcomings of an ERC model. The model has 12 attention heads, 12 hidden layers, and one hidden group. The embedding size and hidden size are set to default with values 128 and 768 respectively. The size of maximum positional embeddings is 160. The vocabulary size is set to 30,000. The input to the model is similar to that given to a BERT model with input ids, masks, and segment ids which are *int32* tensors of shape [*batch_size, max_sequence_length*]. The model handles the input in one token sequence where sequence refers to the input to the ALBERT model which can be a single sentence or multiple sentences. A special token [CLS] is appended at the beginning of every sequence. The final hidden state corresponding to this token is used in the small network to classify the emotion. Multiple sentences in the same token are separated by token [SEP]. A learned embedding token is added to each sequence in order to indicate the context which is passed through subsequent segments and positional embeddings. The preprocessed data is then passed through the pretrained ALBERT model which gives two possible outputs, pooled_output and sequence_output with shapes [*batch_size, hidden_size*] and [*batch_size, max_sequence_length, hidden_size*] respectively. We use the pooled_output of the pre-trained ALBERT model which has a size of [*batch_size, 768*] as the hidden size is 768. The output of the ALBERT model is then passed through a series of three Feedforward layers each with 100 nodes and reLu activation function which was concurred upon through the process of experimentation. The number of feedforward networks started from one and we increased it until the best accuracy was achieved (which was found at three). For the nodes, we started with 20 nodes and increased gradually until no further improvement in the accuracy was seen (in this case 100). Different activation functions were also used, but reLu proved to be the best amongst them. The last layer is a dense layer that contains six nodes, each node to predict that specific emotion with a *softmax* activation function which smooths out our output accordingly on the IEMOCAP dataset. In case of the MELD dataset, the last layer contains seven nodes as there are seven possible emotion outputs.

**Algorithm 1:** Pseudo-code for proposed model.

**Input:** Dataset as *preprocessed*_*D*_*train*_ and *train*_*labels*, *D*_*test*_ and *test*_*labels*
**Output:** Accuracy as *A* and Weighted *F*1 Score as *F1*
1 *model* ← pretrained_model (“*ALBERT*_*en*_*base”*)
// Load ALBERT_en_base pretrained model
2 *no*_*of*_*layers* ← 3
3 **for** i in no_of_layers **do**
4 *model* ← model + *Dense*(*n* = 100; *a*_*f* = “*relu”*; *i*)
/* Adding 3 dense layers with 100 nodes and reLu activation function */
5 **end for**
6 *model* ← model + *Dense*(*n* = 6; *a*_*f* = “*softmax”*)
/* Adding Dense layer with 6 nodes and *softmax* activation function for output */
7 *epoch* ← 4
8 **for** *each e in epoch* **do**
9 *utterance* ← *preprocessed*_*D*_*train*_ (“*batch*_*size* = 1”)
10 *h*_*train*_ ← trainModel (*utterance*)
// Train classification Model
11 **end for**
12 *h*_*test*_ ← runModel(*D*_*test*_, *h*_*train*_)
// Predicting for test data
13 *A* ← calculateAccuracy(*test*_*labels*, *h*_*test*_)
14 *F*1 ← calculateF1Score(*test*_*labels*, *h*_*test*_)
// Calculating Accuracy and weighted F1 score
15 **return** *A*, *F*1

The Emotion classification model itself has 11,771,190 parameters out of which all of them are trainable. The number of epochs is set to four based on a trial and error method. The accuracy and F1-score gradually increase from epoch 1 to 4 with its maximum reaching on epoch four after which the model overfits. The batch_size is set to 1, indicating that we take each sentence in the dataset into consideration to make changes in the parameters before the next step.

#### Computational resources: *compute Canada*

This research was enabled in part by support provided by *Compute Canada* (www.computecanada.ca) For running our codes, we use the *Compute Canada* cluster, running on 14 CPU cores with 30,000 M CPU memory and allocated time for the execution of the whole program as 150 min. The program terminates after 150 min or earlier with the output stored in a file. The GPU specifications used are as follows: Model: P100-PC21E-12GB, with two GPUs per CPU socket.

## Results and Discussion

### Comparison with baseline and state-of-the-art model (IEMOCAP)

For comprehensive evaluation of our model, a comparison is made with the baseline models and state-of-the-art models. We provide a thorough comparison of results mentioned in Table 3 with the baseline-models as well as the state of the art models. We use both the accuracy and Weighted F1-Score for comparison between various models. Following is a brief description of each model used for comparison:

**Table 3:** Comparison of performance of proposed model with baseline models in terms of accuracy and F1-score.

Methods	Happy		Sad		Neutral		Angry		Excited		Frustrated		Average	
	Acc	F1	Acc	F1	Acc	F1	Acc	F1	Acc	F1	Acc	F1	Acc	F1
CNN	27.77	29.86	57.14	53.83	34.33	40.14	61.17	52.44	46.15	50.09	62.99	55.75	48.92	48.18
Memnet	25.72	33.53	55.53	61.77	58.12	52.84	59.32	55.39	51.50	58.30	62.70	59.00	55.72	55.10
CMN	25.00	30.38	55.92	62.41	52.86	52.39	61.76	59.83	55.52	60.25	71.13	60.69	56.56	56.13
DialogueRNN	28.47	36.61	65.31	72.40	62.50	57.21	67.65	65.71	70.90	68.61	61.68	60.80	61.80	61.51
BiDialogueRNN	25.69	33.18	75.10	78.80	58.59	59.21	64.71	65.28	80.27	71.86	61.15	58.91	63.40	62.75
AdCOFE	54.94	54.84	56.69	56.64	61.73	59.68	72.71	73.04	64.11	65.00	69.67	67.12	64.51	64.72

• CNN: The model architecture presented by Kim (2014) is variant of the traditional Convolutional neural networks (CNN) architecture. The feature extractor focuses on capturing the important feature, the one with the highest value obtained from the feature map. The multiple filter feature extractor does not use contextual information and works with variable sentence lengths.

• Memnet: A neural network in the form of a memory network that is trained end-to-end and hence requires less supervision is proposed. The sentences in the model presented by Sukhbaatar et al. (2015) are represented in two different representations. The first approach is a bag of words that embeds each word of the sentence and sums the vectors which in turn fails to capture the importance of keywords. The second approach focuses more on the position of the words wherein element-wise multiplication is performed in order to obtain the positional encoding. The main essence of the model lies in the importance of the temporal context of the sentence. To enable this in the model, the memory vector is modified to encode the temporal information. The output matrix is also embedded the same way. Both the matrices are learned during training, which helps in getting the best possible answer that is the emotion behind the sentences.

• CMN: The proposed conversational memory networks (CMN) model by Hazarika et al. (2018) involves multimodal feature extraction for all utterances involving memory networks. The process of feature extraction involves a fusion of features extracted at three levels-audio, visual, and textual which are joined to form the utterance representation. The memory network is modeled using GRU cells which consider the context information from the history and its preceding utterances. Finally, the dynamics of the model are presented by feeding the current utterance to two distinct memory networks.

• DialogueRNN: Majumder et al. (2018) proposed a system that employs three gated recurrent units focusing on the three major aspects of emotion detection: the speaker, context from preceding emotions, and emotions behind the preceding utterances. The proposed model follows a feature extraction technique similar to CNN followed by the three GRU layers.

• BiDialogueRNN: Majumder et al. (2018) proposed another variant of DialogueRNN which has both a forward pass and a backward pass using RNN. In this variant, the emotion is passed taking into consideration both the previous utterances and the upcoming utterances.

As evident, AdCOFE surpasses the accuracy and F1-score of all the baseline models (CNN, Memnet, and CMN) and the state-of-the-art Dialogue RNN model. In terms of F1 score, AdCOFE surpasses the accuracy of baseline models approximately by 16.5%, 9.6%, and 8.6% respectively. When compared to BiDialogueRNN model AdCOFE exceeds the accuracy and F1 score approximately by 1.1% and 2% respectively.

### Comparison with latest ERC models (IEMOCAP)

The research in the field of emotion recognition has been rapidly increasing and to provide a comprehensive idea of the AdCOFE model’s performance, a comparison of the results is made with the following latest advanced ERC models:

• EmoGraph : The proposed model consists of a graphical neural network that uses co-occurrence statistics in between every emotion class considering each emotion class as a node of the graph. This network then helps in extracting features from neighbours of each emotion node Xu et al. (2020).

• AGHMN: Jiao, Lyu & King (2019) proposed an Attention Gated Hierarchical Memory Network (AGHMN) consisting of a Hierarchical Memory Network (HMN) with a primary focus on the enhancement of features at utterance level and of the contextual information memory bank. Two layers are defined for modeling of word sequence and for capturing historical utterances with Attentions GRU to retain positional information of the utterance.

• RGAT: Ishiwatari et al. (2020) uses positional encodings in the Relation-aware Graph Attention network.

AdCOFE is heavily based on extracting contextual features from the data in contrast to current complex models using graphical and attention based neural network and provide comparable results (Table 4).

**Table 4:** Comparison of F1-score with latest ERC models.

Model	F1 score
EmoGraph	65.4
AGHMN	63.5
RGAT	65.22
AdCOFE	64.7

### Model variants (IEMOCAP)

The following section deals with the different variants of the framework presented in this paper. The results of each variant along with their respective F1 score are displayed in the Table 5 below.

**Table 5:** Accuracy of the model after every step.

Model	Accuracy	F1 score
ALBERT	58.2	58.6
ALBERT + Batch Sentences	58.4	58.7
ALBERT + ConceptNet	62.3	62.7
ALBERT + ConceptNet + VADER	64.1	64

Initially, the fine-tuned data is passed to the ALBERT model. The performance obtained in terms of classification of emotions is low as the model does not consider contextual information. The model treats the data as just lines of conversations and fails to capture high-level contextual information. This results in low accuracy. Further to enhance the accuracy the sentences in the dataset are arranged in specific batches wherein similar sentences of one speaker are clubbed together. This variation focuses on passing on the emotion throughout which in turn improves the accuracy. Since any conversation associates a natural meaning along with it, more focus on the meaning of each word helps us in better classification of emotions. The improvised version of the model makes use of a knowledge graph that captures the natural language meaning behind each word and sentence. The results generated as a result of the ConceptNet API further enhances the contextual information of the utterances. The final variant combines all the above features along with the sentiment lexicon which rates the sentences according to the valence as positive, negative, neutral based on a threshold. This added embedding extensively improves the accuracy. The final product provides us with a framework that overcomes all the disadvantages of the previous old models and majorly focuses on feature extraction and contextual enrichment.

### Comparison with state of the art models (MELD)

Table 6 provides a comparison of the weighted F1-score with some of the state-of-the-art models. As evident, AdCOFE provides comparable and better results as compared with other state-of-the-art models.

**Table 6:** Comparison of accuracy and weighted F1-score.

Model	Accuracy	Weighted F1 score
DialogueRNN	59.540	0.5703
bc-LSTM+Att	57.5	0.5644
KET	–	0.5818
AdCOFE	60.2	0.5871

## Conclusion and Future Work

AdCOFE focuses heavily on extracting contextual features from the data. The human brain is able to comprehend the emotion of the sentences not only based on the words and their relation in the sentence but also because it has vast contextual information from its own memory. We apply the same logic to execute our model by using knowledge graphs for an advanced contextual understanding of the sentences and sentiment lexicons for adding context-based emotional features to the sentences. The implemented model surpasses the accuracies and F1-score for the baseline models and the state-of-the-art model. Furthermore, AdCOFE entirely runs using pretrained models and transfer learning, and results are achieved by running only four epochs, thus making it a simple and elegant model to implement. Overall, AdCOFE implements a unique idea of focusing on context and accomplishes good results when compared with other complex neural network models. For future work, we plan to focus more on enhancing the classification model for passing the emotions of each utterance throughout the conversation in order to capture long-range contextual information more effectively.

## Additional Information and Declarations

### Data Availability

The following information was supplied regarding data availability: The code is available at GitHub: https://github.com/VBhat97/AdCOFE.
